# Improved medical expenditure and survival with integration of traditional Chinese medicine treatment in patients with heart failure: A nationwide population-based cohort study

**DOI:** 10.18632/oncotarget.20063

**Published:** 2017-08-08

**Authors:** Ming-Yen Tsai, Wen-Long Hu, Jen-Huai Chiang, Yu-Chuen Huang, Shih-Yu Chen, Yu-Chiang Hung, Yung-Hsiang Chen

**Affiliations:** ^1^ Graduate Institute of Integrated Medicine, College of Chinese Medicine, Research Center for Chinese Medicine & Acupuncture, China Medical University, Taichung, Taiwan; ^2^ Department of Chinese Medicine, Kaohsiung Chang Gung Memorial Hospital and Chang Gung University College of Medicine, Kaohsiung, Taiwan; ^3^ Management Office for Health Data, China Medical University Hospital, Taichung, Taiwan; ^4^ College of Medicine, China Medical University, Taichung, Taiwan; ^5^ Department of Medical Research, China Medical University Hospital and School of Chinese Medicine, China Medical University, Taichung, Taiwan; ^6^ School of Chinese Medicine for Post Baccalaureate, I-Shou University, Kaohsiung, Taiwan; ^7^ Department of Psychology, College of Medical and Health Science, Asia University, Taichung, Taiwan

**Keywords:** traditional Chinese medicine, heart failure, NHIRD, medical expenditure, survival

## Abstract

**Background:**

No previous studies have evaluated the effects of traditional Chinese medicine (TCM) treatment on patients with heart failure (HF). Hence, in this study, we determined whether TCM treatment affects the healthcare burden and survival of HF patients.

**Methods:**

Samples were retrieved from the registry of catastrophic illness patients of the Taiwan National Health Insurance Research Database (NHIRD). Based on a frequency (1:1) matched case-control design, patients with HF between 2000 and 2010 were designated as cases (TCM users) and controls (non-TCM users). TCM treatment for patients with HF was analyzed.

**Results:**

Among these patients, 312 used TCM for HF treatment and exhibited significantly increased 5-year survival (*p* < .0001), with multivariate adjustment, compared with those without TCM use. Mean outpatient clinic visits at 1 year and 5 years after HF diagnosis were higher in TCM users, and accumulated medical costs were lower than in non-TCM users at 1 year. The hospitalization cost at 1-year follow-up was lower for TCM users than for non-TCM users. We found that, compared with non-TCM users, TCM users had an 86% reduction in risk of mortality in the compensated group, and a 68% reduction in the decompensated group receiving TCM treatment (aHR 0.32, 95% CI 0.20–0.52). The hazard ratio (HR) of Chinese herbal medicine (CHM) users with HF was significantly lower than that of non-users (aHR 0.24, 95% CI 0.16–0.35). We also analyzed the most commonly used herbal products as well as the HRs associated with their use, thus providing future research avenues.

**Conclusions:**

This nationwide retrospective cohort study finds that combined therapy with TCM may improve survival in HF patients. This study also suggests that TCM may be used as an integral element of HF interventions on health care costs.

## INTRODUCTION

Heart failure (HF) is a common cause of morbidity and mortality [1–3]. The prevalence of HF is increasing worldwide due to both an aging population and significant advances in the management of associated comorbidities, such as ischemic heart disease, arrhythmia, diabetes mellitus, stroke, and hypertension [4, 5]. Currently, >5 million American people are affected by HF, and 0.5 million patients are newly diagnosed with HF every year [6].

HF is also a leading use of medical resources, with significant healthcare costs driven by the intensive visits to clinics and extended hospital lengths of stay (LOS) [7]. HF imposes substantial costs, and total annual expenditures in the United States exceed $30.7 billion [8]. A report from the Ministry of Health and Welfare of Taiwan estimated that the total annual expenditure associated with HF was (New Taiwan Dollars) NTD (1 NTD@0.329 U.S. dollars) 7.7 billion in 2009 [9]. Despite notable technological achievements and the exploitation of new drugs over recent decades, HF patients still have poor prognosis, with a 5-year survival rate of 50 % after initial diagnosis [6].

Due to dissatisfaction with conventional western medication in treating HF [10], more and more HF patients are turning to complementary and alternative medicine (CAM) to improve the symptoms and signs of the disease [11]. Traditional Chinese medicine (TCM) is regarded as a CAM therapy in Western countries [12, 13]. However, it is a mainstream therapy in some Asian countries, such as Taiwan, Korea, and China. Some reports have expressed that a holistic approach of TCM could be employed in the treatment of HF [14], with the focus on the correction of overall disharmony of the human body by using multi-targeted herbs to regulate and nourish qi-blood and yin-yang [15, 16].

The National Health Insurance (NHI) is a universal health insurance program established in 1995 in Taiwan. It covers both western medicine and TCM [17]. With a high insured rate of 98-99 %, the random sample that comprises the NHI research database (NHIRD) is representative of the general population and should allow a reasonably accurate assessment of the utilization of medical resources in Taiwan [18]. Therefore, the NHIRD provides an ideal platform for pharmacoepidemiological studies for understanding the benefits of TCM in Taiwan. Approximately 30% of Taiwanese people use TCM [19], and up to 70 % of patients have received TCM for chronic diseases such as ischemic heart disease [20], ischemic stroke [21] and type II diabetes mellitus [22]. Our previous data from NHIRD showed that 78.5 % of patients newly diagnosed with HF visited a TCM outpatient at least once in 2001-2010. The data also indicated the frequency and prescription patterns of Chinese herbal medicine (CHM), which could provide understanding of the practices of TCM practitioners in the treatment of HF [23]. However, no evidence to date has indicated that TCM could play a protective role in treating HF. We therefore conducted a nationwide population-based survey of HF patients to estimate the healthcare burdens of services for those who did and those who did not receive TCM treatment, and to identify the outcomes of the two populations.

## RESULTS

**Figure 1:**
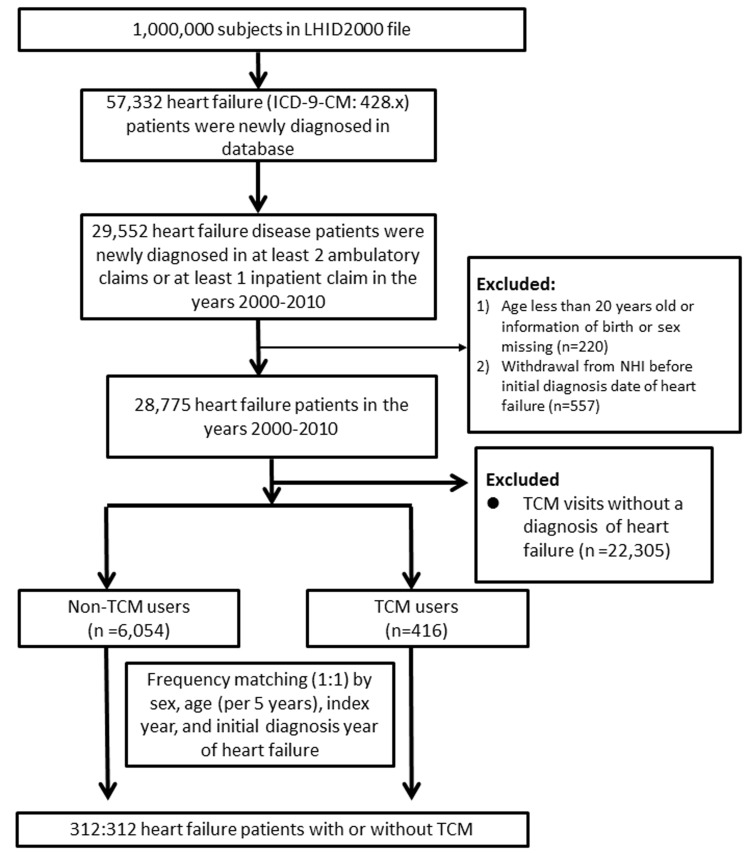
Flowchart of the recruitment of subjects who underwent TCM treatment from the 1 million random samples of the National Health Insurance Research Database (NHIRD) from 2000 to 2010 in Taiwan

From 2000 to 2010, 312 subjects met the criterion of TCM use for HF and were defined as TCM users, and an equal number of matched subjects were classified as non-TCM users. (Figure 1). The characteristics of patients in both groups are listed in Table 1. The percentages of the two groups at different ages were 1.6% in the 20-39 year old group, 34.9% in the 40-64 year old group, and 63.5% in the over 65 year old group. Females were slightly more represented than males. The mean duration of receiving TCM treatment after the diagnosis of HF was 414 days. Regarding the comorbidities of patients with HF, the incidences of most diseases, including hyperlipidemia, chronic obstructive pulmonary disease, and coronary artery disease, were higher in TCM users. In contrast, the incidences of diabetes mellitus and stroke were higher in non-TCM users.

**Table 1:** Characteristics of heart failure patients according to the use of TCM or not

Variable	HF patients				*P* value
	TCM users				
	No (*n* =312, 50%)		Yes (*n* =312, 50%)		
	*n*	%	*n*	%	
**Sex**					0.99^*^
Female	167	53.53	167	53.53	
Male	145	46.67	145	46.47	
**Age group**					0.99^*^
20-39	5	1.60	5	1.60	
40-64	109	34.94	109	34.94	
> 65	198	63.46	198	63.46	
Mean ± SD ( years)	68.51 (12.03)		68.27 (11.88)		0.8028 ^a^
**Co-morbidity**					
Hyperlipidemia	110	35.26	142	45.51	0.0090^*^
DM	133	42.63	99	31.73	0.0049^*^
CAD	141	45.19	166	53.21	0.0453^*^
COPD	127	40.71	145	46.47	0.1462^*^
Stroke	122	39.10	101	32.37	0.0794^*^
**The duration between onset of HF and the first visit to a TCM clinic, days, mean (median)**	-		414 (0)		

^*^Chi-Square test; ^a^ t test

The mean (median) of the follow-up period were 6.28 (5.62) years and 2.33 (0.91) years for TCM user and non-TCM users, respectively.

Abbreviation: TCM, traditional Chinese Medicine; DM, diabetes mellitus; CAD, coronary artery disease; COPD, Chronic Obstructive Pulmonary Disease; SD, standard deviation.

Table 2 compares the clinical visits of outpatients and hospitalization of the TCM users and non-TCM users for periods of 1 and 5 years. TCM users had significantly more outpatient visits than did non-TCM users in the 1 and 5 year periods (49±49 vs. 23±20, p-value < .0001; 188±202 vs. 70±79, *p* <.0001). As compared with the HF without TCM treatment group, TCM users had lower medical expenditures (53,602 ± 75,379 NTD vs. 64,154 ± 140,817 NTD, *p* < 0.001) for outpatient care within the 1-year after HF diagnosis. The medical expenditure for hospitalization in the first year after HF differed significantly between the two groups (*p* =0.0171).

**Table 2:** Clinical visits for outpatient care and hospital length of stay, and their medical costs, for TCM and non-TCM users within 1 year and 5 years after diagnosis of heart failure

Cost	Non-TCM users		TCM users		*P* value^§^
	*n*	Mean (SD)	*n*	Mean (SD)	
**Clinical visits**					
Outpatients care visits	254	23.44 (19.99)	312	49.43 (49.07)	<.0001
Hospital length of stay (days)	130	60.55 (209.06)	96	39.63 (132.70)	0.3597
**Cost**					
Outpatients care, NTD	254	64,154(140817)	312	53,602 (75379)	<.0001
Hospitalization, NTD	130	243,614 (382984)	96	139,888 (265767)	0.0171
**5 year**					
**Clinical visits**					
Outpatients care visits	257	70.12 (78.80)	312	188.08 (202.39)	<.0001
Hospital length of stay (days)	199	134.62(1097.03)	201	108.54 (368.48)	0.7507
**Cost**					
Outpatients care, NTD	257	222,161 (566458)	312	238,376 (427151)	0.7051
Hospitalization, NTD	199	315,813 (548970)	201	337,875 (603776)	0.7025
**Clinical visits**					

Abbreviate: TCM, traditional Chinese medicine; SD, standard deviation

^§^ t-test

Table 3 shows the HR and details of the CHM most frequently prescribed by TCM practitioners for treating HF. The results show that Zhi-Gan-Cao-Tang (3,563 person-days) was the most frequently prescribed formula CHM, followed by Sheng-Mai-San (2,989 person-days) and Zhen-Wu-Tang (1,445 person-days). With regard to the single herbs for HF, Dan Shen (3,559 person-days) was the most common. San Qi (2,079 person-days) and Jie Geng (1,800 person-days) were the second and third most commonly used herbs, respectively. For most patients, receiving CHM was associated with significant reductions in HR.

**Table 3:** HRs and 95% CIs of mortality risk associated with the most-used herbal products as compared with non-CHM group among heart failure patients

Pin yin Nomenclature	Scientific Name	Accumulated Person-Days	*n*	Frequency of Mortality	Hazard Ratio (95% CI)	
					Crude^*^	Adjusted^†^
Non-CHM group			312	268	1.00 (reference)	1.00 (reference)
CHM group			312	44	0.23 (0.16-0.34)^***^	0.24 (0.16-0.35)^***^
**Single herb**						
Dan Shen	*Rx. Salviae Miltiorrhizae*	3,559	63	8	0.22 (0.11-0.44)^***^	0.25 (0.13-0.51)^***^
San Qi	*Radix Notoginseng*	2,079	28	8	0.58 (0.29-1.16)	0.69 (0.35-1.39)
Jie Geng	*Platycodonis Radix*	1,800	26	2	0.16 (0.04-0.62)^**^	0.19 (0.05-0.77)^*^
Fu Zi	*Aconiti Lateralis Preparata*	1,336	50	6	0.22 (0.10-0.49)^***^	0.27 (0.12-0.60)^**^
Zhi Ke	*Fructus Aurantii*	1,334	10	2	0.34 (0.09-1.37)	0.39 (0.10-1.55)
Gui Zhi	*Ramulus Cinnamomi*	1,064	39	4	0.21 (0.08-0.55)^**^	0.24 (0.09-0.63)^***^
Huang Qi	*Rx. Astragali*	988	47	7	0.28 (0.13-0.58)^***^	0.31 (0.15-0.64)^***^
Dang Shen	*Radix Codonopsis*	897	29	5	0.36 (0.15-0.87)^*^	0.35 (0.15-0.85)^*^
Wu Wei Zi	*Fructus Schisandrae*	722	18	3	0.33 (0.11-1.01)	0.40 (0.13-1.23)
Ren Shen	*Rx. Ginseng*	610	10	1	0.14 (0.02-1.01)	0.17 (0.02-1.23)
**CHM formula**						
Zhi-Gan-Cao-Tang	*Rx. Glycyrrhizae Preparata, Rx. Ginseng, Ram. Cinnamomi, Rx. Rehmanniae, Tub. Ophiopogonis, Colla Corii Asini, Sm. Cannabis, Rz. Zingiberis Recens, Fr. Zizyphi Jujube, White Wine*	3,563	73	12	0.28 (0.16-0.49)^***^	0.35 (0.20-0.61)^***^
Sheng-Mai-San	*Rx. Ginseng, Tub. Ophiopogonis, Fr. Schisandrae*	2,989	106	17	0.29 (0.18-0.47)^***^	0.32 (0.20-0.52)^***^
Zhen-Wu-Tang	*Rx. Aconiti Lateralis Preparata, Rz. Atractylodis Macrocephalae, Poria, Rz. Zingiberis Recens, Rx. Paeoniae Alba*	1,959	45	6	0.26 (0.11-0.57)^***^	0.26 (0.12-0.59)^**^
Suan-Zao-Ren-Tang	*Sm. Zizyphi Spinosae, Poria, Rx. Anemarrhenae, Rx. Ligustici, Rx. Glycyrrhizae*	1,445	7	0	-	-
Tian-Wang-Bu-Xin-Dan	*Rx. Rehmanniae, Rx. Ginseng, Rx. Asparagi, Rx. Ophiopogonis, Rx. Scrophulariae, Rx. Salviae Miltiorrhizae, Poria, Rx. Polygalae, Rx. Angelicae Sinensis, Fr. Schisandrae, Sm. Platycladi, Sm. Zizyphi Spinosae, Rx. Platycodi, Cinnabaris*	1,306	36	2	0.09 (0.02-0.37)^***^	0.11 (0.03-0.44)^**^
Xue-Fu-Zhu-Yu-Tang	*Sm. Persicae, Flos Carthami, Rx. Angelicae Sinensis, Rz. Chuanxiong, Rx. Paeoniae Rubra, Rx. Cyathulae, Rx. Bupleuri, Rx. Platycodi, Fr. Aurantii, Rx. Rehmanniae, Rx. Glycyrrhizae*	1,031	37	4	0.19 (0.07-0.50)^***^	0.22 (0.08-0.58)^**^
Ji-Sheng-Shen-Qi-Wan	*Rx. Rehmanniae Preparata, Fr. Corni, Rx. Dioscoreae, Rz. Alismatis, Poria, Cx. Moutan, Cx. Cinnamomi Loureroi, Rx. Aconiti Lateralis Preparata, Rx. Cyathulae, Sm. Plantaginis*	999	33	8	0.46 (0.23-0.93)^*^	0.43 (0.21-0.86)^*^
Bu-Zhong-Yi-Qi-Tang	*Rx. Astragali, Rx. Ginseng, Rz. Atractylodis Macrocephalae, Fried Rx. Glycyrrhizae, Rx. Angelicae Sinensis, Per. Citri Reticulatae, Rx. Cimicifugae, Rx. Bupleuri*	821	30	6	0.39 (0.17-0.86)^*^	0.44 (0.20-0.98)^*^
Si-Ni-Tang	Rx. Aconiti Lateralis Preparata, Rz. Zingiberis, Cx. Cinnamomi, Rx. Glycyrrhizae Preparata	722	23	1	0.09 (0.01-0.66)^*^	0.10 (0.01-0.69)^*^
Liu-Wei-Di-Huang-Wan	*Rx. Rehmanniae Preparata, Fr. Corni, Rx. Dioscoreae, Rz. Alismatis, Poria, Cx. Moutan*	680	15	4	0.52 (0.19-1.39)	0.61 (0.23-1.63)

Crude HR^*^ represented relative hazard ratio; Adjusted HR† represented adjusted hazard ratio: mutually adjusted for age, gender, hyperlipidemia DM, CAD, COPD and stroke in Cox proportional hazard regression.

^*^p < 0.05, ^**^p < 0.01, ^***^p < 0.001

A total of 614 patients diagnosed with HF were classified into two subgroups. A total of 31 death events occurred in 83 patients with decompensated HF who were prescribed TCM, whereas 13 death events occurred in the 229 subjects without decompensation who used TCM after HF diagnosis. We found that, compared with non-TCM users, TCM users in the compensated group tended to have a mortality rate lower than that of TCM users in the decompensated group (Table 4). In the compensated group, the HR of mortality of TCM users was 0.14 times lower than that of non-TCM users. In the decompensated group, patients who received TCM treatment also had a lower risk of mortality (aHR 0.32, with 95 % CI 0.20 − 0.52).

**Table 4:** Hazard Ratios and 95% confidence intervals of mortality risk associated with TCM user among heart failure developing to decompensation or not

	*n*	Mortality	Hazard Ratio (95% CI)	
		no	Crude^*^	Adjusted^†^
Decompensated group				
Non-TCM user	139	54	1 (reference)	1 (reference)
TCM user	83	31	0.39 (0.24-0.61)^***^	0.32 (0.20-0.52)^***^
Compensated group	173	36		
Non-TCM user	229	13	1 (reference)	1 (reference)
TCM user	139	54	0.14 (0.07-0.27)^***^	0.14 (0.07-0.28)^***^

Crude HR^*^ represented relative hazard ratio;

Adjusted HR† represented adjusted hazard ratio: mutually adjusted for age, gender, hyperlipidemia DM, CAD, COPD and stroke in Cox proportional hazard regression.

^*^p < 0.05, ^**^p < 0.01, ^***^p < 0.001

**Figure 2:**
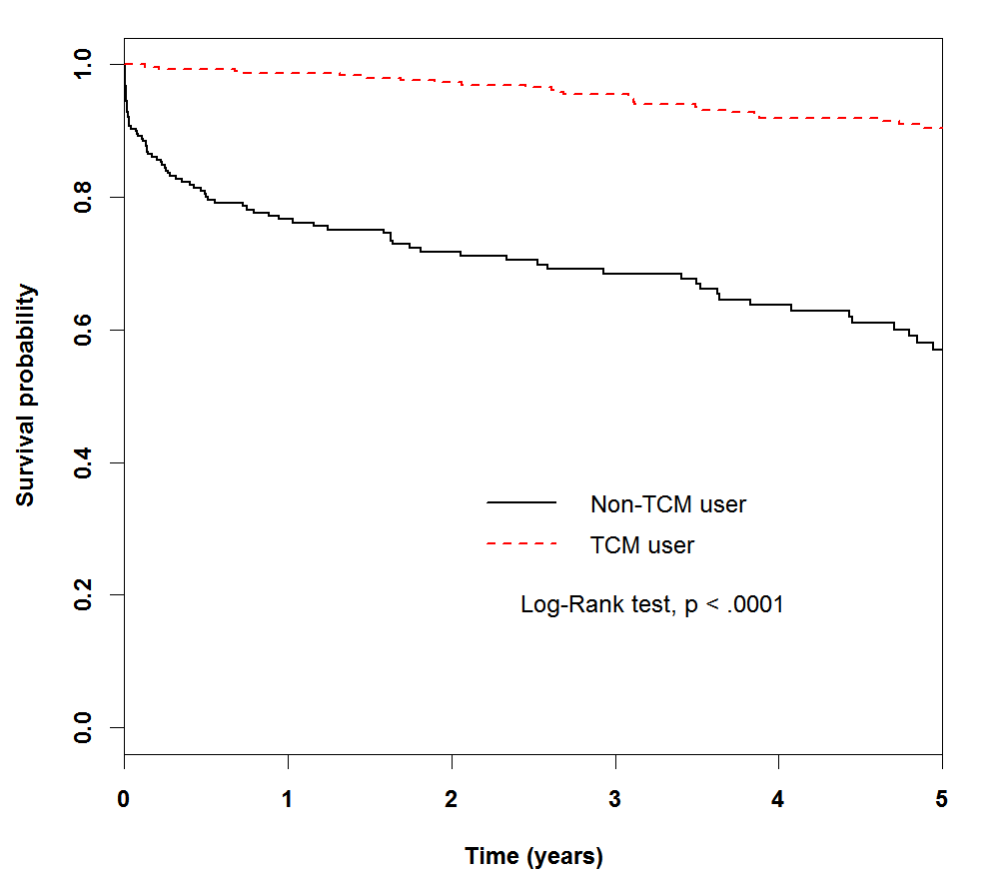
Kaplan-Meier curves of 5-year survival rate in patients with heart failure for TCM users and non-TCM users (5-year survival: non-CHM user, 0.5692; CHM user, 0.9050).

The difference in mortality between the TCM and non-TCM groups was also illustrated through a Kaplan-Meier survival graph, as shown in Figure 2 (*p* < .0001).

## DISCUSSION

This research is the first large-scale study on the economic burden and survival of TCM by patients with HF and was conducted by analyzing claims data from TCM and non-TCM clinic visits covered by the NHI in Taiwan. In a previous study [23], we investigated the frequency and prescription patterns of CHM for HF and found the same three most common CHMs and similar trends in the female gender and younger age groups of patients seeking TCM. However, the proportion of TCM utilization rate in this study is far below the previous one (6.43% vs. 78.5%). This difference is due to the criterial design of the analyzed population for specific diseases in epidemiology or statistics. We knew that many registries in the NHI claims are primarily used for administrative billing and are not verified for scientific purposes [24]. Communicating with data must be done cautiously; for example, some HF patients registered in NHIRD had visited TCM clinics regardless of the real cause of HF. The possible overestimation of the TCM utilization rate is an important issue that needs to be emphasized. In addition, TCM physicians often use the symptoms of the ICD code to replace the patient's real diagnosis because of limitations in the health insurance payment system and medical prescription days. The accuracy of medical coding in the claims may affect the data validity because subjective opinions on diagnoses and prescriptions may vary across individual physicians in different clinical contexts [25].

The widespread uses of mortality-altering medications such as beta-blockers and ACE inhibitors have changed the prognosis of HF [26, 27]. However, the outcome of HF is grim. In a study of over 30,000 patients admitted with new-onset HF, the 1-year mortality rate was 30% [28]. An overview from the Framingham Heart Study, which used 50 years of follow-up data with HF, reported that the 5-year mortality rates in men and women were 59% and 45%, respectively [29]. Our analyses confirmed patients’ diagnoses by using NHI claims and focused on records from outpatient files containing treatment by TCM clinicians. The results showed that only a smaller number of HF patients had received TCM therapy, which was far below the annual utilization rate of other diseases (29-30%) [30]. However, the potential benefits of TCM treatment cannot be excluded. The improvement in survival can be seen in the Kaplan-Meier curve, which clearly illustrates that HF patients in the TCM group had longer survival times.

The majority of studies that have analyzed HF outcomes have focused on HF hospitalizations [31, 32]. HF patients who need admission to use pharmacologic and mechanical circulatory support to restore their compensatory mechanisms are likely to have had severe functional impairment (over 80% in New York Heart Association (NYHA) classes III and IV) [31, 32]. Hospital admission for the intensive treatment of decompensated HF is an unfortunate certainty, regardless of etiology. In-hospital mortality for patients with decompensated HF ranges from 3-4%, increasing to 10% at 90 days [31, 32]. Although our previous study noted that chart-level records are not available in the NHIRD for use as outcome indicators among patients with HF [31, 32], the current study found, from analyzing indirectly the codes of compromised respiratory function, that patients who used TCM treatment after mild to severe HF tended to have lower risk of mortality. Therefore, to reduce mortality, the use of TCM treatment for patients with advanced stage HF could be considered.

In patients with HF, all-cause hospitalization can be as high as 58% during 1-year follow-up and is associated with worse outcome [33]. Moreover, more frequent and longer hospitalizations would increase the inpatient mortality rate over time [34, 35]. The greatest economic burden occurs within the first months after the first HF hospitalization or diagnosis [36], and patients face the highest financial costs of the disease in the first year [37]. Costs with hospitalization for decompensation reach approximately 60% of the total expenditures for the treatment of HF [31, 32]. Therefore, efforts should be made to reduce hospital LOS in order to improve outcomes and decrease medical expenditures. Our study of TCM users and non-users, as measured by the NHI, clearly shows beneficial economic results in lowering inpatient costs during 1-year follow-up.

However, it must be noted that we analyzed the medical expenditures of patients with HF, but not the costs due to the disease itself. Part of the calculated costs might not have been directly related to HF but secondary to the care of other ongoing co-morbidities. In addition, more evidence of TCM—not only hospital LOS, but also death event, re-hospitalization, and general modern lab indexes—should be explored in future research.

Our most significant finding was that the use of CHM was associated with a significant decrease in HR. The HR of the CHM group was 76% lower than that of the non-CHM group. Moreover, it was shown that most of the common CHM formulas or single herbs prescribed by TCM physicians decreased HR. Certain influences, such as the training background of physicians and characteristics of patients, cannot be ruled out in this type of retrospective study; however, this TCM syndrome differentiation and treatment supports the possibility of a causal relationship between the two. The fundamental problem in HF in TCM theory is the prolonged deficiency of heart qi and yang, which reduces the heart's ability to drive blood and transport fluid, leading to blood stasis and excessive fluid retention [38]. Therefore, the therapeutic principles are mainly to invigorate the heart yang-qi—specifically by accelerating blood circulation, liberating flow of the network vessels, and disinhibiting accumulated water [39].

Although clinical research on the effects of TCM on HF patients remains scarce, we propose several possible explanations for why a lower mortality is observed in patients with HF receiving TCM. First, patients with HF who undergo TCM treatment may have better knowledge or vigilance of disease prevention, both of which are considered to be protective factors against disease [40, 41]. This improvement of the medical burden in TCM users in our study strengthens the possibility of a causal relationship between outpatient utilization rate and decreased HR. Second, some potential mechanisms by which CHMs act against HF have been established in previous studies. The potential mechanisms include improvement of cardiac function and histopathologic changes [42], sympathetic activation [43], oxidative stress of cardiovascular injury [44], immunity [45], and neuro-hormonal imbalance [46]. According to the results of this study, some CHMs were found to be beneficial for HF treatment (i.e., Zhi-Gan-Cao-Tang and Zhen-Wu-Tang) and to improve the survival rate of HF patients. Third, patients with HF might have an increased risk of insomnia and sleep-related complications, such as fatigue, depression, and worsening functional performance [47]. Insomnia is a potential risk factor for HF [48], and Suan-Zao-Ren-Tang and Tian-Wang-Bu-Xin-Dan are commonly used in East Asia to improve insomnia and other mood problems [49]. Pharmacological studies have shown that both may exert beneficial effects on HF through stabilizing the central nervous system and reducing sympathetic overactivity [50, 51]. Indirect treatments for sleep disorders may in turn lower the mortality of HF. Finally, according to a published study, more than half of the medical burden is applied to manage HF-related symptoms such as nausea, loss of appetite, fatigue, and general weakness [52]. These symptoms decrease quality of life, lower medical adherence, and reduce the intake of nutrients [53], and they also increase the risk of cardiovascular events [54]. Some CHM drugs for reinforcing qi (i.e., Bu-Zhong-Yi-Qi-Tang, Sheng-Mai-San, *Rx. Ginseng* and *Rx. Astragali*) appear to be effective in improving the clinical sequelae of inadequate cardiac output and inefficient venous return [55–58]. According to a recent review, TCM and natural herbal medicines are promising therapeutic strategies for treating anorexia, malnutrition, or cachexia [59]. Additionally, CHM treatment has been associated with regulation of the catabolic state and cytokine dysfunction by inhibiting TNF-α, IL-6, IL-10, and INF-γ production [60]. Thus, by reducing the symptom burden in patients with HF through TCM treatment, clinical events including emergency department visits, hospitalizations, and long-term mortality in HF patients may be decreased.

Several limitations must be considered. First, the NHI program contains records for only CHMs prescribed by TCM physicians, not over-the-counter CHMs such as folk medicine and herbal decoction. Therefore, the utilization use of TCM may have been underestimated. Second, we used the ICD-9-CM codes recorded by clinicians to identify HF, but we had no physical symptom, radiological, or biochemical data to account for the severity of HF. Grading the severity of HF by additional codes of respiratory distress may produce overlaps with other common medical conditions, particularly COPD, and fail to detect worsening of HF symptoms without pulmonary edema. These shortcomings of the data can be overcome in future studies with the use of ICD-10-CM Terminology. Third, data on adverse drug reactions in this retrospective study were lacking, so we were unable to evaluate the safety of TCM. In addition, much evidence has been reported on drug-herb interactions. For example, Zhen-Wu-Tang, Si-Ni-Tang, and *Aconiti Lateralis Preparata* may increase digoxin toxicity [61], *and Rx. Salviae Miltiorrhizae* may interact with the anticoagulant action of warfarin [62, 63]. However, such interactions seemed not to affect the clinical CHM prescription in our study. Fourth, we were unable to confirm whether the benefits of CHM were a direct result of the CHM used, a synergistic effect with cardiovascular drugs, or even just a result of the CHM reducing the adverse effects and consequently leading to a more successful prognosis. More epidemiological data on safety outcomes should be collected to clarify the causality in future studies.

In conclusion, this nationwide cohort study reveals that TCM treatment lowered the risk of death in patients with HF. Our findings also show that TCM appeared to reduce the healthcare system costs for HF. Study of the CHM formulas and single herbs commonly used to treat HF is warranted in higher-quality, randomized-controlled trials to validate these observational findings. The use of TCM is worthy of attention and further research.

## MATERIALS AND METHODS

### Data sources

Taiwan's compulsory universal NHI program was developed in 1995 by the National Health Insurance Administration, and it initially provided coverage to more than 23.03 million residents of Taiwan. By 2008, >99% of the Taiwanese population was enrolled in the NHI program. Reimbursed TCM services include CHM, acupuncture, moxibustion, or manipulation in ambulatory clinics. The database contains all longitudinal reimbursement information, as well as gender, birth date, medications, and diagnosis codes based on the International Classification of Disease, Ninth Revision, Clinical Modification (ICD-9-CM). This study was designed as a population-based study of a sample of one million subjects selected at random from the 22 million beneficiaries of the National Health Insurance scheme of Taiwan. The sampled patients exhibited no significant differences in age, sex, birth year, or average insured payroll-related amount from the general population. Because the NHIRD contains delinked secondary data for research, the need for informed consent was waived for this study. This study was approved by the Institutional Review Board of China Medical University (CMUH104-REC2-115).

### Study population

All patients 20 years old and above diagnosed with HF (ICD-9-CM: 428) between January 2000 and December 2010 were included. Inclusion criteria for the population with HF (n=29,552) were at least two ambulatory visits or one inpatient claim with diagnosis of ICD-9-CM code 428 from 2000 to 2010. Patients were excluded if they were lost to follow-up in the NIH program for >1 year or had received TCM treatment before the initial date of HF but not in the tracking period. Patients having at least one medical record in a TCM outpatient clinic and the HF diagnostic code were defined as TCM users, whereas those who had no TCM outpatient records were defined as non-TCM users. For each TCM user, a control subject who was a non-TCM user was randomly selected by frequency-matching (1:1) with the case cohort to ensure that both had the same distributions for the strata of sex, age (per 5 years), and the time from the date of the first HF diagnosis to the index date.

### Covariate assessment

The sociodemographic factors included age and sex. Age was divided into 3 groups: 20-39 years old, 40-64 years old, and >65 years old. Baseline comorbidities were considered present if ICD-9-CM codes appeared in at least two ambulatory claims or one inpatient claim before the initial diagnosis date of HF, included diabetes mellitus (ICD-9-CM: 250), hyperlipidemia (272), coronary artery disease (410-414), chronic obstructive pulmonary disease (COPD; 490, 491, 492, 494 and 496), and stroke (430-438). Decompensated HF, defined as a sudden or gradual worsening of HF signs and symptoms, is usually caused by rapid fluid accumulation in the lungs necessitating further hemodynamic and oxygen support [61]. Therefore, pulmonary edema (518.4) and respiratory failure (518.81) were selected to represent the decompensated state.

### Variables for expenditures and claim visits

The database contained encounter forms based on the date of visit, medical facility visited, department visited, type of copayment, and billed and paid amounts. All visits within 1 year and 5 years after the index date were analyzed for expenditures as well as outpatient and inpatient claim visits.

### Statistical analysis

Differences in demographic characteristics and comorbidities between the study and comparison cohorts were examined using the Chi-squared test for categorical variables and 2-sample t tests for continuous variables. The means of outpatient and inpatient visits and medical expenditures of TCM users and non-TCM users were compared with 2-sample t tests. In the future analysis, the risks of mortality for decompensated HF and compensated HF were calculated for TCM and non-TCM users. A univariate and multivariate Cox proportional hazards model was used to evaluate the hazard ratios (HRs) of TCM users on mortality. We estimated hazard ratios and their 95% confidence intervals (CIs) by adjusting for age, gender, hyperlipidemia, DM, CAD, COPD, and stroke in Cox proportional hazard model regression.

Statistical analysis was performed and figures were created using SAS 9.4 (SAS Institute, Cary, NC) software. P < 0.05 in two-tailed tests indicated statistical significance.
